# Child Mortality Inequalities and Linkage with Sanitation Facilities in Bangladesh

**Published:** 2008-03

**Authors:** Amal K. Halder, M. Kabir

**Affiliations:** 1 Department of Statistics, Jahangirnagar University, Dhaka 1342, Bangladesh; 2 Health Systems and Infectious Diseases Division, ICDDR,B, GPO Box 128, Dhaka 1000, Bangladesh; 3 Department of Statistics, Jahangirnagar University, Dhaka 1342, Bangladesh

**Keywords:** Child mortality, Concentration index, Health equity, Inequalities, Infant mortality, Mortality, Neonatal mortality, Principal component analysis, Sanitation, Socioeconomic status, Bangladesh

## Abstract

Principal component analysis (PCA) was applied to assets and other household data, collected as part of the Bangladesh Demographic and Health Survey (BDHS) in 2004, to rank individuals according to a household socioeconomic index and to investigate whether this predicts access to the sanitation system or outcomes. PCA was used for determining wealth indices for 11,440 women in 10,500 households in Bangladesh. The index was based on the presence or absence of items from a list of 13 specific household assets and three housing characteristics. PCA revealed 35 components, of which the first component accounted for 18% of the total variance. Ownership of assets and housing features contributed almost equally to the variance in the first component. In this study, ownership of latrines was examined as an example of sanitation-intervention access, and rates of mortality of neonates, infant, and children aged less than five years (under-five mortality) as examples of health outcomes. The analysis demonstrated significant gradients in both access and outcome measures across the wealth quintiles. The findings call for more attention to approaches for reducing health inequalities. These could include reforms in the health sector to provide more equitable allocation of resources, improvement in the quality of health services offered to the poor, and redesigning interventions and their delivery to ensure that they are more pro-poor.

## INTRODUCTION

The international community is committed to the Millennium Development Goals (MDGs), most of which are closely related to health status or determinants of health (1). The MDGs have been widely accepted as a framework for improving health and welfare worldwide. Child mortality is one of the most crucial preventable global health concerns. In many low-income countries, 10–20% of children die before reaching five years of age (compared to, for example, 0.7% in England and Wales) (2). The MDG relating to child mortality (to reduce the under-five mortality rate by two-thirds between 1990 and 2015) was formulated as a national average. The World Health Report 2003 posed an important question: how does progress towards the MDGs affect equity? We investigated this by examining, across a range of settings, how inequality in under-five mortality of the poorest and the richest changes as progress is made towards the MDG.

The relationship between socioeconomic status and health has been an area of increasing interest (3,4). The relationship between the socioeconomic status differentials and the health status in developing countries has been documented in several studies (5-10). Using demographic and health survey data from Ghana, Gwatkin *et al*. described the differences between the poor and the least poor in mortality, nutrition, and treatment of illness (9). Woelk and Chikuse, in Zimbabwe, showed that stunting, underweight, and occurrence of diarrhoea varied according to socioeconomic status, noting that being in the lowest socioeconomic group increased the risk of being underweight for children by about three times compared to those in the highest socioeconomic group (10). In a study of 22 malarious countries in Africa, Filmer found little difference in rates and risk of fever among the poor and least poor, but the poorest had a much smaller likelihood of obtaining suitable treatment (4). A Bangladesh Equity Watch paper evidenced that, although there was some progress in reducing the infant mortality rate and maternal mortality ratio since the independence of Bangladesh in 1971, the health of the disadvantaged section of the population (in terms of geographical location, socioeconomic status, gender, etc.) has not improved as much as it has for the better-off groups (11).

The basic purpose of the study reported here is to describe the socioeconomic status differentials in relation to the status of under-five mortality and access to sanitation services using data from the Bangladesh Demographic and Health Survey (BDHS) 2004. A socioeconomic status index, based on household assets, housing conditions, and facilities of water and fuel utilities, was made using principal component analysis (PCA). This was used for determining the relationship between household socioeconomic characteristics and inequalities of access to sanitation intervention and to health outcomes in Bangladesh. This study specifically examined how proxies for socioeconomic status, e.g. ownership of assets, housing quality, water, and cooking fuel, relate to mortality in neonates, infants, and under-five children and ownership of latrines, a preventive sanitation intervention. The results obtained are intended to assist programme providers and policy-makers to recognize inequalities in the sanitation system to improve the under-five mortality status in Bangladesh.

## MATERIALS AND METHODS

The BDHS 2004 is the fourth survey of this type in Bangladesh. The fieldwork, commenced on 1 January 2004, was completed on 25 May 2004. The BDHS 2004 was conducted under the authority of the National Institute for Population Research and Training (NIPORT), the Ministry of Health and Family Welfare, Government of Bangladesh. Mitra and Associates, a Bangladeshi research firm, implemented the survey. ORC Macro of Calverton, Maryland, USA, provided technical assistance to the project as part of its international demographic and health survey programme, and the U.S. Agency for International Development (USAID)/Bangladesh provided financial assistance.

The BDHS 2004 sample is a stratified, multistage cluster sample consisting of 361 primary sampling units—122 in urban areas and 239 in rural areas. After receiving permission, the BDHS 2004 dataset was taken from the Internet (www.measuredhs.com). The dataset includes information about 11,440 eligible women recorded from 10,500 households. The data file consists of information on background characteristics, such as age, education, religion, etc., socioeconomic information, reproductive history, family-planning methods, antenatal and delivery care, breastfeeding and weaning practices, vaccination and health of under-five children, marriage, fertility preferences, causes of death of under-five children, and so on. These households (10,500) had consistent data for assets, housing conditions, and water and sanitation variables sufficient to create a household socioeconomic status index.

Weighted data were analyzed using the SPSS software (version 11.5). Using the SPSS software, PCA was applied to socioeconomic data to obtain an index as a proxy for household socioeconomic status.

PCA [PCA transforms the original set of variables into a smaller set of linear combinations that account for most of the variance of the original set] involves breaking down assets (radio, bicycle, motor cycle, television, wardrobe, table, chair/bench, watch or clock, cot or bed, sewing machine, ownership of any homestead, ownership of any land, hygienic latrine, floor-wall-roof material) and household access to service (e.g. water, electricity, cooking fuel) into categorical or interval variables. The variables were then processed to obtain weights and principal components. The results obtained from the first principal component [The first principal component of a set of variables is linear index of all the variables that captures the largest amount of information that is common to all the variables (31)] (explaining the most variability) are usually used for developing an index based on the formula:

A_j_=f_1_x(a_ji_-a_1_)/(S_1_)+ ………. + f_N_x(a_jN_-a_N_)/(S_N_) (12)

Where f_1_ is the scoring factor or weight or coefficient (determined by using PCA in the SPSS program) for the first asset (or service); x is the variable (asset or service); a_j_ is the value for assets (or service); and a_1_ and S_1_ are the mean and standard deviation of assets (or service) respectively. Based on this equation, socioeconomic status of the households was assigned, and the resulting population was divided into socioeconomic status quintiles. The quintiles developed were, thus, expressed in terms of quintiles of individuals of the total population at risk for all measures. The 1^st^, 2^nd^, 3^rd^, 4^th^, and 5^th^ quintiles were assigned in the continuum of the poorest and the richest respectively.

Three statistical indicators of inequality were measured. One was the richest/poorest ratio which is the ratio comparing the rate prevailing in the richest quintile with the rate in the poorest quintile. This method ignores information contained in the middle three quintiles. The second measure used was the concentration index calculated by the method of Kakwani *et al*. (13). This measures the extent to which a variable is distributed unequally across all the five socioeconomic status quintiles, i.e. the concentration of inequality. The closer the index to zero is, the less concentrated the distribution of inequality (9). The third measure was a trend test (chi-square) to determine the significance of any gradient in the inequality.

The health-status indicators used by this study were mortality of children aged less than five years (neonatal, infant, and under-five mortality), and the sanitation-intervention indicator is household ownership of latrine. Although the under-five mortality status for the previous 10-year period preceding the survey was described briefly in the BDHS 2004 (14), this study has almost the similar look for the recent five-year period, and the study has, moreover, established a linkage between under-five mortality and a sanitation-intervention indicator. Examining the relationship between the index proposed and these indicators serves as a way of testing the consistency of the index with other data that are possibly related to socioeconomic status. The differentials in health-related indicators according to socioeconomic status groupings would imply that the index is sensitive to differences.

## RESULTS

### Socioeconomic status index

**Fig. 1:**
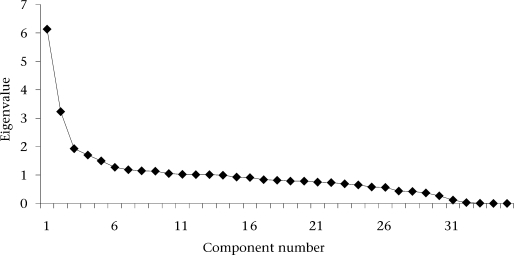
Scree plot—principal component analysis

The final index was based on household assets, housing quality, access to utilities, such as water, and cooking materials and constituted the independent variable. About 18% of the total variance was accounted by the first eigenvalues, i.e. first principal component (Fig. 1, Scree graph), while the rest 34 components captured the remaining 82% variability. The wealth index was constructed exclusively based on the first principal component because it was assumed that the first principal component is a measure of economic status (15).

**Fig. 2:**
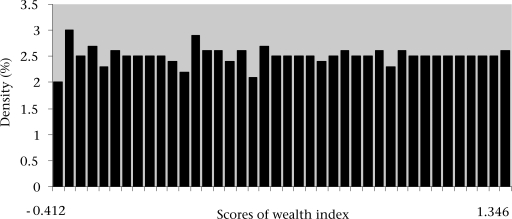
Distribution of scores of wealth index

McKenzie highlights that a major challenge for PCA-based asset indices is to ensure that the range of asset variables included is broad enough to avoid problems of ‘clumping’ and ‘truncation’ (16). Clumping or clustering is described as households being grouped together in a small number of distinct clusters. Truncation implies a more even distribution of socioeconomic status, but spread over a narrow range, making differentiating between socioeconomic groups difficult, e.g. not being able to distinguish between the poor and the very poor. The assumption that the distribution of socioeconomic status is quite uniform is appropriate in this setting. Figure 2 shows a histogram of the household socioeconomic scores for the BDHS 2004 asset variables. The distribution of scores tends to follow a normal curve highlighting no clumping and truncation, which have made it easy to differentiate between socioeconomic groups.

The asset scores (the scores are generated in a table when we give run PCA using the SPSS software [version 11.5]) are then used for assigning a wealth index value to each household and its members. Eventually, households and their members are assigned into quintiles based on the value of the asset index. For the purpose of this analysis, the lowest quintile was considered as a socioeconomic status (wealth index) proxy for the poorest, and the highest quintile represents the richest households.

### Distribution of index components by socioeconomic status

The proportion of households possessing a given characteristic according to the socioeconomic status of that household revealed additional interesting results (Table 1). In general, the poorest were far below the average in most items or services to which the better-of had access. For example, in terms of ownership of assets, 92% of the richest had access to electricity compared to 3% of the poorest. The same applied to radio, TV, bicycle, motorcycle, etc. Thus, as expected, the better-off were likely to own more assets than the poorest.

**Table 1:** Distribution of assets by quintiles

Variable	SES quintiles (% of population)						Ratio (richest/ poorest)
	Poorest	2nd	3^rd^	4th	Richest	Average	
The respondent woman has at her household							
Electricity	2.9	13.1	34.4	67.4	91.6	41.9	32
Radio	6.1	18.9	32.8	53.3	61.2	34.5	10
Television	0.0	1.2	7.2	41.3	79.3	25.8	
Bicycle	4.8	19.1	31.7	40.2	35.6	26.3	7
Motor cycle	0.0	0.2	0.4	1.0	10.5	2.4	
Almirah (wardrobe)	1.1	5.4	22.1	53.6	75.8	31.6	69
Table	10.6	58.0	80.0	90.5	89.9	65.8	8
Chair/bench	13.2	62.0	81.9	90.6	92.1	68.0	7
Watch or clock	14.1	58.9	84.3	94.0	97.4	69.8	7
Cot or bed	74.4	93.2	97.6	99.0	98.8	92.6	1
Sewing machine	0.1	1.0	3.2	6.8	18.7	6.0	187
Owns any homestead	89.1	93.5	96.1	97.7	95.5	94.4	1
Owns any land	26.8	50.3	60.3	67.7	61.9	53.4	2
Drinking-water sources							
Piped inside dwelling	0.0	0.1	1.3	1.3	24.1	5.4	
Piped outside dwelling	0.2	0.5	1.3	2.1	5.2	1.9	26
Tubewell	95.5	93.3	91.0	89.2	64.5	86.7	1
Deep tubewell	1.2	2.3	3.6	3.8	3.8	3.0	3
Surface well/other well	1.0	1.2	1.1	0.5	0.7	0.9	1
Pond/tank/lake	1.4	1.8	1.1	1.9	0.8	1.4	1
Other-Shallow tubewell, river/stream, etc.	0.7	0.7	0.4	0.8	0.5	0.6	1
Living house made of							
Floor (*kancha*)	99.9	99.6	98.8	95.9	22.8	83.4	0
Floor (wood)	0.0	0.0	0.7	1.0	0.7	0.5	
Floor (cement/concrete)	0.0	0.0	0.1	2.4	76.1	15.7	
Wall (jute/bamboo/mud (*kancha*)	79.6	63.3	48.8	32.0	4.1	45.5	0
Wall (wood)	0.4	1.4	2.4	3.0	0.8	1.6	2
Wall (brick/cement)	0.1	1.1	3.5	11.3	77.1	18.6	771
Wall (tin)	20.0	33.9	45.0	53.3	17.7	34.0	1
Roof (*kancha*-bamboo/thatch)	21.1	10.2	5.0	2.0	0.5	7.8	0
Roof (tin)	78.3	87.9	92.5	94.7	63.3	83.3	1
Roof (cement/concrete)	0.6	1.7	2.3	3.0	35.8	8.7	60
Uses							
Cooking fuel: LPG/natural gas	0.1	0.7	1.0	1.8	30.7	6.9	307
Cooking fuel: firewood, straw	12.8	21.5	35.6	51.2	44.0	33.0	3
Cooking fuel: dung	6.1	8.5	10.8	8.4	4.2	7.6	1
Cooking fuel: crop residue/grass	78.3	64.2	43.5	26.7	7.2	44.0	0
Cooking fuel: other—electricity, biogas, kerosene	0.1	0.2	0.4	0.4	2.6	0.8	26

SES=Socioeconomic status

These observations are consistent with the directions of the scores. Like ownership of assets, housing conditions tended to reflect the economic status of the households. A similar pattern was noted for the sources of energy for cooking. Households that ranked lower in the index were more likely than the better-off to use cooking fuel: crop residue/grass (the richest/poorest ratio was almost zero), water from tubewell, and had *kancha* floor material. The congruence between the socioeconomic status index and the variables from which it was generated provided evidence of internal consistency of the developed index.

### Socioeconomic status and health outcomes: mortality of children

*Distribution of neonatal mortality* [Neonatal death: Neonatal death is the term for babies who die within 0–28 day(s) (four weeks) of birth]

In total, 286 neonatal deaths were registered during 1999–2003. Table 2 shows data on how neonatal mortality is distributed across the different socioeconomic status quintiles. The findings showed a significant inverse trend such that the neonatal mortality rate declined with increase in the socioeconomic status of the household. Neonatal children in the poorest households were about 24% (richest/poorest ratio=1.24, and the chi-square trend was statistically significant) more likely to die than those in the richest or better-off. If the socioeconomic status of the poorest households were improved to the level of the better-off, about nine lives (rate difference) per 1,000 neonatal babies could be saved during the reported period (1999–2003).

**Table 2:** Child mortality by socioeconomic status, 1999–2003

SES quintile	No. of births	No. of neonatal deaths	No. of infant deaths	No. of under-five deaths	Neonatal mortality rate (95% CI)	Infant mortality rate (95% CI)	Under-five mortality rate (95% CI)
1^st^ (poorest)	1,807	82	135	160	47 (37–57)	76 (63–89)	90 (77–104)
2^nd^	1,482	55	85	106	36 (26–46)	57 (45–68)	71 (57–85)
3^rd^	1,394	64	99	115	48 (36–60)	74 (59–88)	86 (71–101)
4^th^	1,211	40	56	64	33 (22–43)	45 (32–57)	51 (38–64)
5^th^ (richest)	1,169	45	75	78	38 (27–50)	63 (49–77)	66 (52–80)
Poorest-richest ratio					1.24	1.20	1.37
Concentration index					−0.037	−0.047	−0.070
Chi-square trend					p<0.001	p<0.001	p<0.001

CI=Confidence interval; SES=Socioeconomic status

*Distribution of infant mortality* [Infant mortality is the death of infants in the first year of life]

The relationship between socioeconomic status and infant mortality is shown in Table 2. As with neonatal mortality, socioeconomic status had some association with child mortality, with the poorest households having higher probabilities of child death than the richest. Also similar to the relationship found in neonatal deaths, the pattern was not consistent between the second and the third quintile as neonatal, infant and under-five mortality was higher in the third quintile than in the second quintile. The reasons for this inconsistency are not known but may be due to differences in the heterogeneity of scores within quintiles.

*Distribution of under-five mortality* [Under-five mortality rate: The number of children, aged less than five years, who die in a year, per 1,000 livebirths during the year]

The relationship between socioeconomic status and under-five mortality is summarized in Table 2. The data presented indicate that under-five mortality was higher in the poorest quintile and lower for the rest of the quintiles. The pattern was very similar to that of neonatal deaths. The data revealed that children of the poorest quintile were 37% more likely to die before reaching their fifth birthday than those of the richest quintile households. The gradient of under-five mortality was not consistent between the second and the third quintile. The under-five mortality in the poorest households had similar inequitable poor-richest risks of dying as the neonatal mortality. This indicates that the differentials noted at neonatal mortality have shaped the relationship between socioeconomic status and under-five mortality. If the socioeconomic status of the poorest households were improved to the level of the richest, about 24 lives (rate difference) per 1,000 under-five children could be saved during the reporting period.

### Socioeconomic status and ownership of latrine

The relationship between socioeconomic status and ownership of latrine is presented separately in Table 3. The results indicated consistently that non-ownership of latrine was associated with the lower socioeconomic quintiles. While only 29% of the poorest women owned latrines, the proportion was more than three times higher for the richest. The poorest/richest ratio of non-ownership of 6.5 revealed that there was a large inequality between the poorest and the richest in terms of ownership of latrine in Bangladesh (the chi-square trend was statistically significant).

**Table 3:** Ownership of latrine by socioeconomic status

SES quintile	No. of women	No. of women not having latrine	% of quintile population without latrine
1^st^ (Poorest)	2,284	1,614	71
2^nd^	2,293	1,236	54
3^rd^	2,274	900	40
4^th^	2,300	531	23
5^th^ (richest)	2,289	261	11
Poorest-richest ratio			6.45
Concentration index			−0.301
Chi-square trend			p<0.001

SES=Socioeconomic status

However, the value (−0.331) of the concentration index showed that the concentration curve L(p) lay above the line of equality, further indicating disproportionate concentration of ownership of latrines among the poor. Similar disproportionate results were revealed in the case of neonatal mortality (concentration index=−0.037), infant mortality (concentration index=−0.047), and under-five mortality (concentration index=−0.070).

## DISCUSSION

Inequalities within countries are almost as significant as inequalities between them (17). To investigate the inequalities within the country, many studies have used and recommended the wealth index approach (9,12,18-20). In a study conducted in several states of India, Filmer and Pritchett found that the asset index significantly correlated with the state headcount index and the domestic product per-capita distributions (12).

A limitation of this study was the lack of a defined variable representing economic status in Bangladesh. The sociocultural perceptions of wealth in specific communities need to be examined further. Given that the current approach to measuring socioeconomic status is largely dependent on the use of household possessions, it will be relevant to understand the importance communities attach to the possession of various items (21). This will be useful in determining which items to include in constructing a wealth index relevant in both rural and urban contexts. This, we hope, will put us in a better position to contribute towards monitoring the impact of national policies aimed at reducing inequalities. Another limitation of the study was that an exact count of [incidence of] neonatal deaths (age at death 0–28 day(s)) was not possible since age at death has been recorded in the database by month only, i.e. if the child died before 30 days, the age was 0 (zero) month.

During analysis, we undertook to check the internal consistency of the wealth index developed by investigating its distribution against the quintiles of the household variables. Outcomes of the investigation disclosed an expected relationship on how the asset and household variables change with the quintiles. And this was noted for all the variables (Table 1), with the exception of using tubewells as a source of safe water, housing materials (specific example, *kancha* floor material, wall material made of bamboo/mud/straw, and *tin* as roof material), and cooking fuel as crop residue/grass where the poorest had a higher percentage than the richest. Using tubewell as a source of safe water was mostly happening in rural areas commonly among people in the poorest quintile. Similarly, poor housing condition, such as floor material and wall material, and cooking with crop residue/grass was the highest among people in the poorest quintile. As a whole, the index developed appears to be practical in capturing some form of material well-being at the household level.

The consistent use of hygienic latrines has been shown to protect individuals from enteric diseases (22). In Bangladesh, hygienic latrines are not available free of charge. As cost is involved in the purchase of sanitary latrines, the most socially-disadvantaged groups may not have much access to the protective effect of hygienic latrines. Accordingly, Table 3 revealed that there was a strong association between the wealth index and the ownership of latrine.

The results of the study showed that both water supply and availability of sanitary facilities had a strong association with child mortality, even after controlling for the effects of the socioeconomic and geographical variables (23). Using quintiles generated from the PCA, the investigation has shown that the poorest have the highest mortality rate compared to the other quintiles. Similarly, results of analysis revealed that ownership of latrine was wholly inadequate in households of the poorest quintile and that there were profound inequalities in access to adequate sanitation in Bangladesh. However, the comparisons between the poorest and the richest ratio for mortality (neonatal=1.24, infant=1.20, and under-5=1.37) and non-owning of latrines (6.5) addressed that, although the length of variation between mortality ratios (poorest/richest) and non-owning latrine ratio was wide, both were in the same direction, which indicates that the sanitation facilities have a strong association with child mortality.

**Fig. 3:**
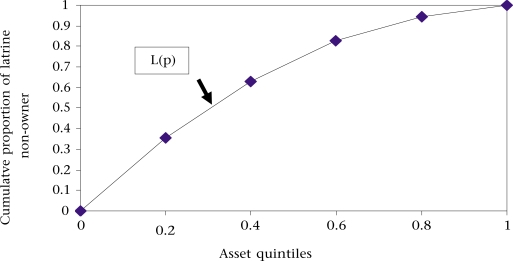
Concentration curve

The values of the concentration indices are another feasible factor in the analysis as the concentration indices showed the degree of inequality across the socioeconomic quintiles. The concentration index is defined as twice the area between the concentration curve (Fig. 3) and the line of equality (the 45^0^ running from the bottom-left corner to the top-right) (24). So, in the case where there is no health-related inequality, the concentration index is zero. The convention is that the index takes a negative value when the curve lies above the line of equality, indicating disproportionate concentration of the health variable among the poor. If the health variable is a ‘bad’ one, such as non-ownership of latrine or child mortality, a negative value of the concentration index means that non-ownership of latrine (Table 3) or mortality (Table 2) is higher among the poor. This is because the analysis evidenced that all the values of the indices were negative. Using the concentration index, a similar result for under-five children in a study demonstrated that a gap exists in health status between the poorest and the richest (25).

In Bangladesh, for neonatal mortality, socioeconomic inequalities increased over time (26). In our study, the neonatal mortality rates ranging from 38 to 47 were much lower than 53.5 per 1,000 livebirths during 1992–1993 (27), but high socioeconomic inequalities were observed. And similar inequalities were clear in the case of infant and under-five mortality. Inequalities in health impact across the quintiles specifically indicated that the health-sector strategies in Bangladesh that reached particularly to poorer quintiles were below than the requirement. Therefore, the findings of the investigation call for more attention to strategies or approaches for reducing health inequalities particularly for the poor. In doing so, health ministries might work more closely with other ministries, but should also take a wider view, e.g. exploring alternative delivery methods to reach the poor and finding improved ways of increasing knowledge among the poor about healthy behaviours (28). These could include reforms in the health sector to provide more equitable allocation of resources, improvement in the quality of health services offered to the poor, and redesigning interventions and their delivery to ensure that they are more pro-poor. Such a proactive measure will be important if health-equity goals at the community level are to be achieved (29).

In showing that a reduction in under-five mortali-ty, in line with the MDG, may result in worsening inequalities in mortality between the poorest and the richest group in society, this study has important policy implications. Since progress towards the MDG may be achieved at the expense of health equality across society, we believe that monitoring under-five mortality among different socioeconomic groups is of the utmost importance (2). Specifically, the MDG relating to child mortali-ty should be reformulated to incorporate an equity dimension, and this would provide an impetus to adopt policies that addressed health inequalities. Also, evidence suggests that a targeted approach has the potential to significantly raise access to health services in Bangladesh (30).

Finally, the study has shown that the BDHS operations can host manageable asset surveys and that a PCA approach to such data is surprisingly sensitive to differences in socioeconomic status. These gradients are sufficient to predict differences in health outcomes, such as child mortality and access to sanitation interventions.

## ACKNOWLEDGEMENTS

This paper is a part of the principal author's PhD thesis. The authors express gratitude to MEASURE DHS, Macro International Inc., Calverton, MD, USA, for providing access to the data of the ‘Bangladesh Demographic and Health Survey 2004’ for this publication. The authors are grateful to Ms Unnati Rani Saha, Data Manager, HSID, ICDDR,B, for her contribution and effective suggestions during principal component analysis. They also expressed their special gratefulness to Dr. Stephen P. Luby, Head of PIDVS, HSID, ICDDR,B, because being a non-statistician he put potential efforts to read thoroughly and made excellent comments to make the study understandable for non-statisticians.
